# Anesthesia-related factors in the pathogenesis of postoperative cognitive dysfunction: a mechanistic perspective

**DOI:** 10.3389/fneur.2025.1700911

**Published:** 2026-01-05

**Authors:** Meng-ni Qin, Ya-nan Deng

**Affiliations:** Department of Anesthesiology, Honghui Hospital, Xi’an Jiaotong University, Xi'an, Shaanxi, China

**Keywords:** postoperative cognitive dysfunction, cognitive decline, anesthesia, neuroinflammation, perspective

## Abstract

Postoperative cognitive dysfunction (POCD) is a significant complication resulting from interactions between anesthesia-related neural disturbances and pre-existing vulnerability. This perspective delineates three major mechanisms: neuroinflammatory activation, oxidative mitochondrial injury, and impaired synaptic plasticity. These interconnected pathways collectively disrupt neuronal homeostasis and contribute to cognitive decline. Clinically, anesthetic choice influences risk, with volatile agents showing stronger neurotoxic potential, whereas dexmedetomidine provides anti-inflammatory benefits. Evidence-based strategies—such as processed electroencephalogram-guided titration, regional anesthesia to reduce opioid use, and cerebral oxygenation monitoring—have demonstrated measurable reductions in POCD incidence. Integrating these findings into a two-hit framework highlights anesthesia as a secondary insult superimposed on age-, frailty-, or metabolism-related vulnerability. Diabetes mellitus exemplifies this first-hit state by creating chronic neuroinflammation, mitochondrial dysfunction, and blood–brain barrier impairment that heighten susceptibility to perioperative stress. Future progress requires precision approaches, including genetic and biomarker-based risk stratification and mechanism-targeted neuroprotective therapies. Methodological limitations—such as heterogeneous assessments and underpowered studies—necessitate standardized multicenter trials with harmonized cognitive testing and extended follow-up. This perspective provides an integrated model of POCD pathogenesis and outlines priorities for advancing individualized perioperative neuroprotection.

## 1 Introduction

Postoperative cognitive dysfunction (POCD) is a central nervous system (CNS) complication that involves deficits in memory, attention, information processing speed, and executive function after surgery (1). Standard diagnostic criteria define POCD as typically emerging within 1 week to several months after surgery; in some cases, it may progress to long-term cognitive impairment or dementia (2). Epidemiological studies show a notably higher incidence of POCD in elderly patients, especially those aged 65 years or older. Rates reach 20–40% among cardiac surgery patients and approximately 10–25% in those undergoing major non-cardiac surgery (3, 4). Despite advances in anesthesia and surgical techniques, POCD continues to pose a major clinical challenge.

The impact of POCD on patient outcomes is multifaceted. For the individual, it can hinder postoperative recovery, raise the risk of falls, extend hospital stays, and significantly reduce quality of life (5). Socioeconomically, patients with POCD often require increased use of healthcare resources and long-term care, which places a considerable financial strain on both families and society (6). Furthermore, emerging longitudinal studies suggest that POCD could be an early sign or may accelerate the progression of neurodegenerative diseases, highlighting the need for deeper investigation into its underlying mechanisms.

Contemporary research in anesthesiology demonstrates that anesthetic management plays a complex and pivotal role in the pathogenesis of POCD. Different anesthetic techniques, namely general anesthesia (GA) and regional anesthesia (RA), exert distinct effects on cognitive function (7, 8). GA agents act directly on the central nervous system by crossing the blood–brain barrier. In contrast, RA, although it avoids the direct central effects of GA, can still affect brain function due to surgical stress responses and hemodynamic instability (8, 9). Clinical studies indicate that GA is generally associated with a higher short-term incidence of POCD compared to RA, although this difference often attenuates in long-term follow-up (9).

Commonly used anesthetic agents have diverse effects on neurological function. The intravenous anesthetic propofol, for instance, allows for rapid induction and recovery but can impair memory formation through potentiation of GABAergic neurotransmission (10). Similarly, benzodiazepines—frequently used for premedication—also enhance GABAergic inhibition and are associated with an increased risk of postoperative delirium (11). However, a direct causal link between benzodiazepines and long-term POCD remains unconfirmed (12). Inhaled anesthetics like sevoflurane enable stable maintenance of anesthesia but may promote neurodegeneration by enhancing *β*-amyloid aggregation and tau hyperphosphorylation (13, 14). In contrast, the α2-adrenergic receptor agonist dexmedetomidine has attracted interest due to its potential neuroprotective effects, although its long-term impact on cognition needs more clinical evidence (15). Importantly, the cognitive effects of anesthetics are typically dose- and time-dependent, and are significantly influenced by the patient’s preoperative cognitive status.

This study examines the role of anesthesia in the development of POCD by pursuing three primary objectives: (1) to characterize the effects of anesthetics on neuroinflammation and synaptic plasticity pathways, (2) to compare cognitive outcomes following different anesthetic techniques in elderly surgical patients, and (3) to evaluate potential neuroprotective strategies. It aims to advance the mechanistic understanding of anesthesia-related neurotoxicity and to develop evidence-based approaches for mitigating POCD risk in vulnerable populations.

**Figure 1:**
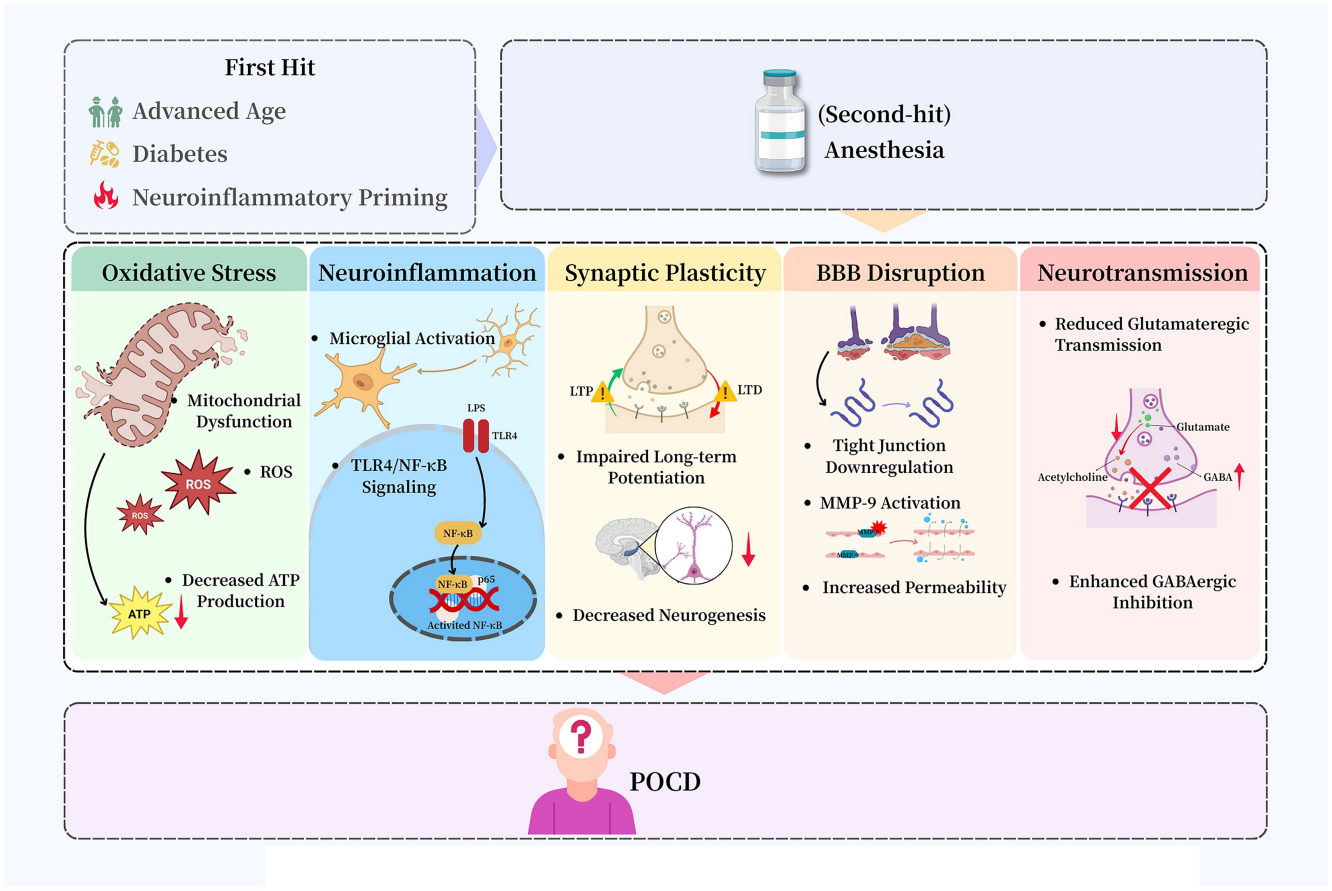
Modular illustration of the two-hit model in which pre-existing vulnerability (“first hit”) interacts with anesthesia as a “second hit,” triggering downstream mechanisms—including neuroinflammation, oxidative stress, neurotransmission imbalance, blood–brain barrier dysfunction, and impaired synaptic plasticity—that collectively contribute to POCD.

This perspective synthesizes current evidence within the conceptual framework of the “two-hit” model of POCD (Figure 1). In this model, the “first hit” consists of a pre-existing neural vulnerability, which is driven by factors such as advanced age, metabolic syndrome, or genetic predisposition, and sensitizes the brain. The “second hit,” represented by the perioperative period, subsequently triggers and amplifies specific pathophysiological cascades that lead to overt cognitive dysfunction. This framework advances beyond the attribution of POCD primarily to anesthesia, instead characterizing the role of anesthesia as a critical potentiating factor within susceptible physiological systems. The model integrates disparate pathophysiological mechanisms into a coherent framework that accounts for clinical heterogeneity and provides a mechanistic basis for personalized care strategies. Building on this framework, this perspective critically evaluates existing evidence, identifies limitations in current prevention paradigms, and proposes a translational roadmap to guide the implementation of risk-stratified perioperative strategies.

## 2 Potential mechanisms of anesthesia-related factors in POCD pathogenesis

The pathogenesis of anesthesia-associated POCD involves multiple interconnected pathways that collectively disrupt neural homeostasis. These mechanisms—including neuroinflammation, oxidative stress, and synaptic dysfunction—do not operate in isolation but exhibit significant crosstalk that amplifies neural injury. For instance, neuroinflammatory signaling can exacerbate oxidative stress, while mitochondrial failure impairs the energy-intensive processes underlying synaptic plasticity. A comprehensive understanding of POCD therefore requires analyzing these pathways as an integrated network. The following sections detail these core mechanisms within the conceptual framework of the “two-hit” model, wherein a perioperative insult is superimposed upon a pre-existing susceptible neural substrate (Figure 1).

### 2.1 Neuroinflammatory response

**Figure 2:**
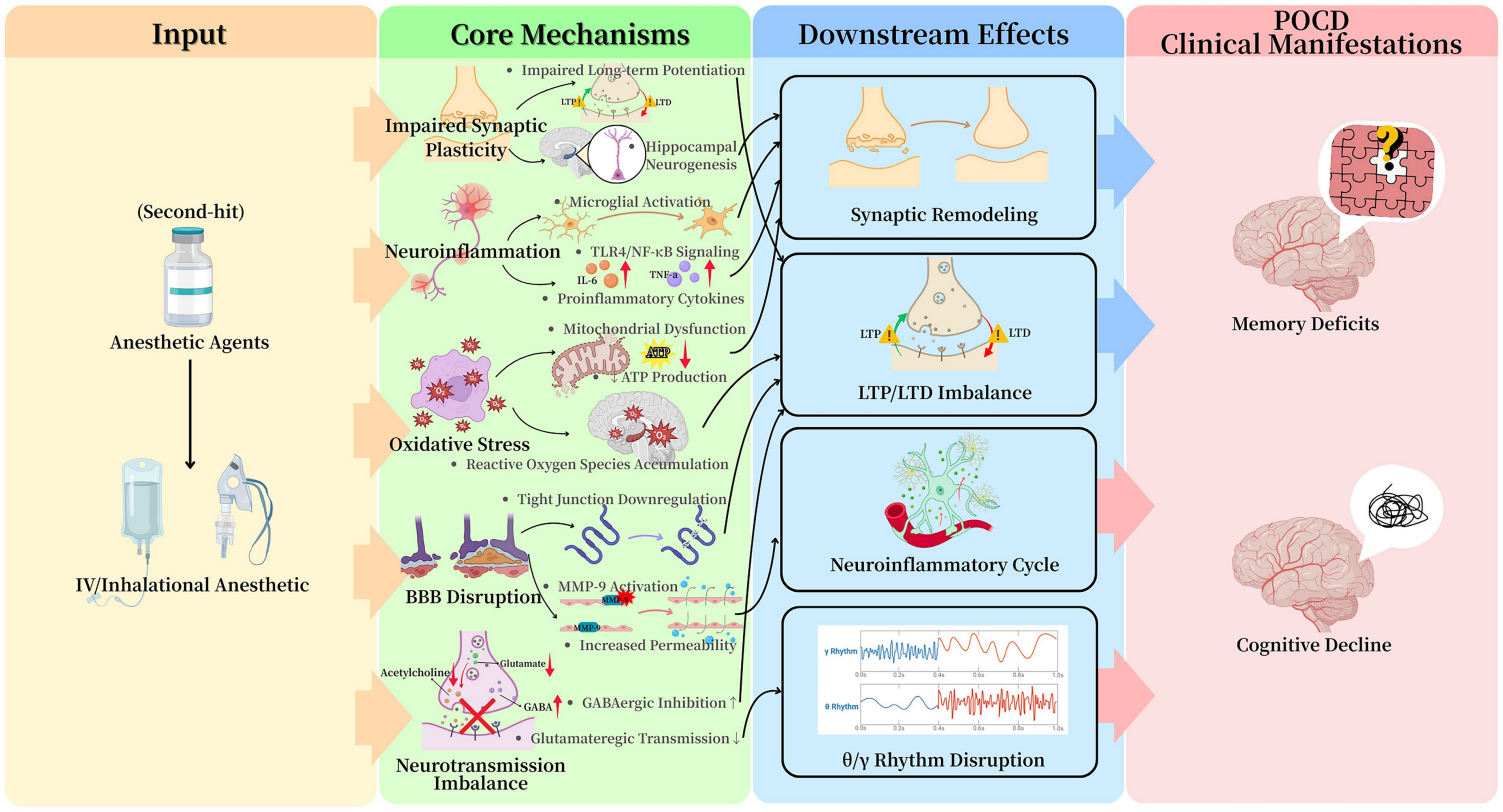
Summary of anesthesia-related mechanistic pathways involved in POCD. Anesthesia acts as a “second hit” that activates five interrelated mechanisms—neuroinflammation, oxidative stress, blood–brain barrier dysfunction, neurotransmission imbalance, and impaired synaptic plasticity. These pathways converge to disrupt neuronal homeostasis and ultimately lead to POCD.

Anesthetic agents are potent modulators of central nervous system inflammation, primarily through microglial activation. Both intravenous and inhalational anesthetics trigger a morphological transition of microglia from a ramified surveillance state to an activated amoeboid form (16, 17). This activation is frequently mediated via the toll-like receptor 4/nuclear factor kappa-B signaling pathway, leading to upregulated transcription and release of pro-inflammatory cytokines including interleukin (IL)-6 and tumor necrosis factor-*α* (TNF-α) (16, 18, 19). The critical role of interleukin-1 beta (IL-1*β*) is demonstrated by studies where intracerebroventricular administration of an IL-1*β* receptor antagonist mitigates sevoflurane-induced cognitive deficits in rodents (20). This anesthetic-induced neuroinflammatory environment can cause acute neuronal dysfunction and potentially initiate a self-sustaining cycle of inflammation that contributes to persistent cognitive decline (Figure 2).

### 2.2 Oxidative stress, mitochondrial dysfunction, and metabolic impairment

Anesthetic-induced oxidative stress represents a fundamental pathway in POCD etiology (Figure 2). Volatile anesthetics and high-dose propofol infusions disrupt mitochondrial electron transport chain function, particularly at Complexes I and III, leading to electron leakage and excessive reactive oxygen species (ROS) generation (21). The resulting redox imbalance depletes endogenous antioxidants, causing oxidative damage to cellular components including lipids, proteins, and mitochondrial DNA. The hippocampus demonstrates particular vulnerability to this damage due to its high metabolic demand and relatively limited antioxidant capacity (22).

Concurrently, anesthetics perturb mitochondrial calcium (Ca^2+^) homeostasis, promoting Ca^2+^ influx into the mitochondrial matrix (23). Elevated matrix Ca^2+^ levels triggers opening of the mitochondrial permeability transition pore, culminating in collapse of the mitochondrial membrane potential, organellar swelling, and release of pro-apoptotic factors such as cytochrome c (23). Furthermore, anesthesia disrupts mitochondrial dynamics by upregulating fission protein Drp1 while suppressing fusion mechanisms, resulting in fragmented, inefficient mitochondrial networks incapable of meeting neuronal energy demands (24). The consequent bioenergetic crisis—characterized by depleted adenosine triphosphate (ATP) production—impairs fundamental neuronal functions including maintenance of ion gradients and synaptic transmission, thereby compromising neural network integrity (25).

### 2.3 Blood–brain barrier (BBB) disruption

Anesthesia can compromise BBB integrity through several identified mechanisms: (1) transcriptional downregulation of tight junction proteins (e.g., claudin-5, occludin), (2) matrix metalloproteinase-9 (MMP-9)-mediated degradation of the basal lamina, and (3) alterations in endothelial cell glucose metabolism (26, 27) (Figure 2). The stabilization of hypoxia-inducible factor-1α under anesthetic conditions enhances MMP-9 expression, directly linking perioperative metabolic stress to BBB breakdown (28). This dysfunction exhibits regional specificity, with the hippocampus and prefrontal cortex showing the greatest increases in permeability. The compromised barrier facilitates the central nervous system influx of peripheral inflammatory mediators, which further amplifies neuroinflammation via toll-like receptor 4/nuclear factor κappa-B signaling pathways (29). The correlation between the time course of BBB recovery and cognitive improvement in animal models underscores its pathophysiological significance in POCD.

### 2.4 Neurotransmitter system dysregulation

General anesthetics induce widespread dysregulation of central neurotransmitter systems. Their primary action involves the potentiation of GABAergic inhibition via positive allosteric modulation of GABAA receptors, leading to prolonged inhibitory postsynaptic currents and desynchronization of neural networks essential for cognition (30). Benzodiazepines, acting at specific GABAA sites, exacerbate this phasic inhibition. This enhanced inhibition can profoundly suppress neuronal activity in hippocampal and cortical networks (31). These effects outlast the anesthetic period, as evidenced by the delayed recovery of gamma-band oscillations, which are critical for cross-cortical information integration.

In parallel, anesthetics suppress excitatory neurotransmission. Volatile agents reduce presynaptic glutamate release probability in hippocampal synapses and postsynaptically inhibit *N*-Methyl-d-Aspartate receptor currents (32). Neurotransmitter synthesis, synaptic vesicle loading, and postsynaptic receptor activation are all highly energy-demanding processes (33). Consequently, mitochondrial dysfunction and the resulting ATP depletion disrupt synaptic transmission by depriving these essential processes of the necessary energy (34). These combined effects substantially impair synaptic plasticity, notably long-term potentiation in hippocampal Cornu Ammonis 1 (CA1) neurons, a cellular cornerstone of memory.

The cholinergic system is notably vulnerable, with microdialysis studies showing 50–70% reductions in extracellular acetylcholine during anesthesia (35). This suppression disrupts hippocampal-prefrontal theta coherence (4–8 Hz), a rhythm vital for working memory and attention. The convergence of these inhibitory-excitatory and neuromodulatory disturbances creates a neurochemical environment highly predisposed to cognitive deficits (Figure 2).

### 2.5 Synaptic plasticity and neurogenic impairment

Prolonged anesthetic exposure directly impairs synaptic integrity and hippocampal neurogenesis (Figure 2). Quantitative analyses show a 30–50% reduction in dendritic spine density in CA1 neurons, alongside decreased levels of key synaptic scaffolding proteins (36). Oxidative stress potentiates these structural deficits by oxidizing and inactifying cytoskeletal regulators like cofilin, promoting actin depolymerization and spine collapse. Functionally, this manifests as an elevated threshold for LTP and a heightened propensity for long-term depression (37), imbalances linked to dysregulated brain-derived neurotrophic factor (BDNF)/proBDNF signaling and protein phosphatase 1 activity (38).

Concurrently, anesthesia suppresses hippocampal neurogenesis, evidenced by a decreased population of doublecortin-positive neural progenitors in the subgranular zone (39). The combination of impaired synaptic plasticity and dampened neurogenesis creates a persistent neural environment conducive to cognitive decline. The strong correlation of these cellular changes with deficits in spatial and recognition memory tasks in animals suggests a direct mechanistic link to the clinical presentation of POCD (39).

### 2.6 The impact of metabolic comorbidities: focusing on diabetes mellitus (DM)

Pre-existing metabolic comorbidities, particularly DM, significantly elevate the risk and severity of POCD (40) (Figure 1). Patients with DM exhibit a 1.5–2-fold higher incidence of POCD compared to non-diabetic individuals (40). This heightened vulnerability stems from the synergistic interaction between the underlying diabetic state and the physiological stress induced by anesthesia and surgery, which concurrently disrupts several pathophysiological pathways (41).

DM perpetuates a chronic state of low-grade systemic and neuroinflammation, characterized by elevated pro-inflammatory cytokines and primed microglia (42). In this sensitized state, subsequent anesthetic or surgical stimuli provoke an exaggerated neuroinflammatory response, leading to accelerated neuronal injury and more severe cognitive deficits (19). Furthermore, chronic hyperglycemia exacerbates oxidative stress through multiple mechanisms, including enhanced mitochondrial ROS production and the accumulation of advanced glycation end products (AGEs) (43). The binding of AGEs to their receptor (RAGE) on glial cells further amplifies oxidative stress and pro-inflammatory signaling (44). Consequently, the diabetic brain demonstrates a reduced antioxidant capacity, increasing its susceptibility to the additional oxidative burden imposed by anesthetics.

Mitochondrial dysfunction represents a pathological hallmark common to both DM and POCD. In diabetes, hyperglycemia and insulin resistance collectively impair electron transport chain function, suppress mitochondrial biogenesis, and promote fission over fusion (45). These alterations result in inefficient and fragmented mitochondrial networks. The administration of anesthetics within this compromised metabolic milieu readily precipitates bioenergetic failure and initiates apoptotic signaling. Concurrently, cerebral insulin resistance and impaired insulin signaling, particularly within the hippocampus, contribute significantly to this vulnerability (46). Insulin acts as a crucial neuromodulator that promotes synaptic plasticity, supports neuronal survival, and facilitates amyloid-beta (A*β*) clearance (47). Dysfunctional insulin signaling in DM compromises these vital processes, thereby increasing the brain’s susceptibility to the synaptotoxic effects of anesthesia (47). The BBB is also impaired in DM, as evidenced by the downregulation of tight junction proteins and increased RAGE expression (48). This BBB dysfunction facilitates the influx of peripheral inflammatory mediators and A*β* into the central nervous system during the perioperative period.

The pathophysiology of DM provides a clear paradigm for the “two-hit” model of POCD. The chronic low-grade inflammation, metabolic dysregulation, and vascular impairment that characterize DM constitute the “first hit,” establishing a state of heightened neural vulnerability (40). The perioperative phase then serves as the “second hit,” integrating the combined stresses of anesthesia, surgery, and potential hemodynamic instability (41). This secondary insult overwhelms the brain’s diminished homeostatic capacity, substantially increasing the risk and severity of POCD. This example illustrates that the risk of POCD is not determined exclusively by intraoperative events but is profoundly influenced by the patient’s preoperative physiological reserve.

## 3 Anesthetic management strategies to mitigate POCD risk

### 3.1 Anesthetic drug selection and cognitive outcomes

Comparative studies demonstrate significant differences in neurocognitive effects between anesthetic agents. Meta-analyses reveal volatile anesthetics increase POCD risk by 15–25% compared to propofol total intravenous anesthesia (TIVA) in elderly patients, particularly for procedures >2 h (49) (Supplementary Table S1). This difference stems from volatile agents’ stronger suppression of hippocampal neurogenesis and promotion of *β*-amyloid aggregation. However, propofol exhibits dose-dependent neurotoxicity, with infusion rates >100 μg/kg/min causing mitochondrial dysfunction through electron transport chain disruption (50), and prolonged use risking propofol infusion syndrome (51).

Recent large-scale clinical trials provide stronger, more generalizable evidence on the cognitive outcomes of different anesthetic techniques. A prospective, multicenter study by Radtke et al. involving more than 1,000 non-cardiac surgery patients found that propofol-based TIVA was associated with a significantly lower incidence of POCD 1 week after surgery compared to volatile anesthetics (52).

The role of benzodiazepines in the pathogenesis of POCD remains a subject of ongoing clinical relevance and debate. Preoperative benzodiazepine administration, especially in elderly patients, is a well-established risk factor for postoperative delirium (53). Delirium is an acute confusional state that frequently precedes or co-occurs with POCD. Proposed mechanisms underlying this link involve potent and prolonged enhancement of GABAergic inhibition, which can cause network hypersynchronization and disrupt communication between the cortex and hippocampus (54). Additionally, benzodiazepines may exert anticholinergic effects that further disrupt the balance of neurotransmitter systems. However, establishing a direct causal link between benzodiazepine use and POCD itself—distinct from delirium—has proven more complex. This effort is confounded by several factors, including the underlying conditions requiring treatment (e.g., anxiety), the specific clinical indications for benzodiazepine use, and concomitant polypharmacy (55). Consequently, clinical guidelines, such as those from the American Geriatrics Society, strongly recommend avoiding benzodiazepines for premedication in older surgical patients (53). When anxiolysis is essential, the use of reduced doses or alternative agents, such as melatonin receptor agonists, is recommended to minimize these risks. This prudent approach reflects a broad clinical consensus: although POCD does not develop in all patients who receive benzodiazepines, their use substantially elevates the risk in vulnerable individuals (53). Therefore, their application must be both judicious and personalized.

The evidence regarding anesthetic modulation of cognitive outcomes must be interpreted in the context of recent large, pragmatic randomized controlled trials. The ENGAGES trial demonstrated that EEG-guided anesthesia, which aims to avoid burst suppression, did not lower the incidence of postoperative delirium compared with usual care (56). The RAGA trial, which compared regional with general anesthesia for hip fracture surgery, also observed no difference in delirium rates (57). Collectively, current evidence establishes that modifying isolated intraoperative factors—such as anesthetic choice or depth—produces negligible effects on postoperative neurocognitive disorders (56, 57). This inefficacy stems from the multifactorial nature of these conditions, where patient vulnerability, surgical stress, and postoperative management collectively determine outcomes. These findings substantiate our central thesis: POCD pathogenesis involves physiological processes extending far beyond anesthetic management. Consequently, we advocate a paradigm shift from seeking universal anesthetic solutions toward implementing integrated perioperative care bundles. Future advances will require preoperative identification of high-risk patients and delivery of tailored intervention packages—a strategic approach we will detail in the final section.

Evidence from high-quality trials consistently demonstrates that targeting a single intraoperative factor—for instance, the choice of anesthetic agent or the depth of anesthesia—rarely succeeds in meaningfully altering the complex, multifactorial course of postoperative neurocognitive disorders (PND), especially delirium (56). This limitation arises because the development of PND is driven by a confluence of factors, including patient-specific vulnerabilities (such as preexisting cognitive decline or frailty), the magnitude of the surgical insult and the subsequent inflammatory response, and various postoperative challenges (like pain, sleep disruption, and polypharmacy) (58). Thus, the pathogenesis of PND involves mechanisms that extend far beyond the realm of anesthetic management. Consequently, a paradigm shift is warranted: moving away from the quest for universal anesthetic solutions and toward the implementation of bundled, multi-modal care pathways designed to support vulnerable patients throughout the entire perioperative continuum.

Dexmedetomidine emerges as a neuroprotective adjunct, reducing early POCD incidence by 30–40% when combined with other agents (59, 60). Its α2-mediated mechanisms attenuate neuroinflammation while preserving cerebral autoregulation. Similarly, a prospective trial by Deiner et al. focusing on elderly surgical patients found that intraoperative administration of dexmedetomidine was associated with improved postoperative cognitive trajectories, supporting its potential neuroprotective role (61). Current evidence supports a balanced approach using low-dose propofol with dexmedetomidine while minimizing volatile exposure, particularly for cognitively vulnerable patients (60). This strategy optimizes anesthesia efficacy while mitigating neurocognitive risks.

### 3.2 Anesthesia depth monitoring and cognitive protection

Electrophysiological monitoring of anesthetic depth significantly influences postoperative cognitive outcomes, especially in elderly surgical patients. Evidence shows that prolonged deep anesthesia—defined by a bispectral index below 40—is an independent predictor of cognitive decline. Each additional hour of deep anesthesia increases the risk of POCD by 18% (62). This dose–response relationship may result from excessive suppression of thalamocortical networks and disruption of functional connectivity essential for cognition (63). Current guidelines recommend maintaining BIS values between 45 and 60 during anesthetic maintenance, as this range balances surgical needs with neural protection.

Emerging monitoring technologies provide enhanced capability for cognitive risk assessment. Entropy monitoring demonstrates particular value in detecting burst suppression patterns, which serve as electrophysiological markers of excessive anesthetic depth and predict subsequent cognitive impairment (64). Clinical implementation studies show that processed electroencephalogram monitoring adoption reduces delirium incidence by facilitating more precise anesthetic titration (65). However, optimal application requires individualized interpretation of monitoring parameters in conjunction with patient-specific factors, as rigid protocolized approaches may inadvertently increase total anesthetic exposure (63).

### 3.3 Multimodal anesthesia and analgesia in POCD prevention

Multimodal approaches combining RA with GA significantly reduce systemic anesthetic requirements by 30–50% while potentially lowering POCD risk (66). Regional techniques, including neuraxial blocks and peripheral nerve catheters, effectively decrease opioid consumption by 40–60% and attenuate surgical stress responses, thereby maintaining more favorable cerebral inflammatory profiles (67). These benefits are particularly evident in major abdominal and thoracic surgeries, where opioid-sparing regimens incorporating non-steroidal anti-inflammatory drugs and local anesthetic infiltration demonstrate 25% lower POCD rates at 3-month follow-up compared to traditional opioid-based protocols (68).

The neuroprotective mechanisms of multimodal analgesia involve three key pathways: (1) reduction of *μ*-opioid receptor-mediated hippocampal apoptosis, (2) prevention of blood–brain barrier disruption from cytokine surges, and (3) preservation of physiological sleep architecture during postoperative recovery (69). Preclinical models confirm that preoperative non-steroidal anti-inflammatory drug administration significantly attenuates neuroinflammation and improves cognitive outcomes, supporting their early incorporation into analgesic regimens (69).

Current best practices recommend individualized multimodal strategies based on surgical complexity and patient risk factors. The most effective approach combines preemptive RA with scheduled non-opioid analgesics, particularly for elderly patients and those undergoing prolonged procedures (67, 68). This evidence-based paradigm shift toward opioid minimization and regional technique utilization represents a significant advancement in perioperative neuroprotection.

### 3.4 Perioperative cerebral oxygenation and hemodynamic management

Cerebral oxygen desaturation (rSO_2_ < 75% baseline) occurs in approximately 40% of major surgeries and significantly correlates with POCD development (70). Near-infrared spectroscopy (NIRS) monitoring enables early detection of these events, with intervention protocols demonstrating 50% reductions in both desaturation duration and associated cognitive decline (71). This monitoring is particularly valuable for elderly patients and those with preexisting cerebrovascular disease, populations most vulnerable to cerebral hypoperfusion.

Optimal hemodynamic management represents a critical neuroprotective strategy. Evidence indicates that mean arterial pressure below 65 mmHg may induce watershed ischemia in vulnerable brain regions, particularly when cerebral autoregulation is impaired (72). Current best practice emphasizes individualized blood pressure targets based on continuous autoregulation monitoring when available, as this approach maintains cerebral perfusion within patient-specific optimal ranges (73). In cardiac surgery patients, such optimization strategies have achieved 35–45% reductions in delayed neurocognitive recovery (74).

The most effective cerebral protection protocol integrates three components: (1) preoperative cerebrovascular reserve assessment, (2) intraoperative NIRS monitoring with protocolized interventions, and (3) maintenance of cerebral perfusion pressure through goal-directed fluid and vasopressor management (74). These measures are particularly crucial for high-risk procedures including cardiac and major vascular surgeries, where they significantly mitigate POCD risk while maintaining hemodynamic stability.

## 4 Future perspectives and challenges in POCD research

To strengthen the translational relevance of these mechanistic pathways, it is essential to clarify their mapping onto clinically observed postoperative cognitive trajectories. Evidence shows that elevated perioperative inflammatory cytokines (e.g., IL-6, TNF-*α*) correlate with impairments in attention and executive function during the first postoperative week (75). In contrast, persistent mitochondrial dysfunction and elevated biomarkers of neuronal injury (e.g., neurofilament light chain, tau) are associated with delayed neurocognitive recovery one to 3 months after surgery (76). Similarly, disruptions in synaptic plasticity and cholinergic neurotransmission have been linked to deficits in working memory and processing speed, as measured by standardized neuropsychological tests (77). Integrating these distinct mechanisms with specific cognitive outcomes clarifies the multifactorial nature of POCD and helps explain the heterogeneity observed among patients.

While current strategies provide a foundation for mitigating POCD, substantial limitations remain. Overcoming these challenges and translating novel discoveries into practice requires focused advancement in several key areas. We propose shifting from uniform interventions to a precision medicine framework consistent with the “two-hit” model of POCD. Priority directions include developing personalized risk stratification and tailored interventions, advancing targeted therapeutics, and implementing methodologically robust clinical studies (76, 78). Progress in these domains will be essential for meaningfully reducing the burden of this complex condition.

### 4.1 Genomic medicine for personalized anesthesia in POCD prevention

Current evidence indicates that genetic factors substantially contribute to the risk of POCD. The ApoE ε4 allele is a key genetic marker, with twin studies demonstrating significantly greater cognitive decline in ε4 carriers after anesthesia exposure (79). This association is likely mediated by impaired amyloid-*β* clearance and cerebrovascular dysfunction, underscoring the role of genetic predisposition in POCD pathogenesis.

Genetic polymorphisms significantly influence individual responses to anesthetic agents. Variants in GABAA receptor subunits, such as GABRA1 and GABRB2, affect sensitivity to propofol and its neurocognitive impact (80). Meanwhile, CYP2B6 polymorphisms alter the pharmacokinetics of anesthetic drugs, prolonging exposure (81). Of particular clinical relevance is the IL-6 rs1800795 polymorphism, which is associated with prolonged postoperative neuroinflammation and persistent cognitive impairment (82).

Beyond ApoE ε4, recent research has identified other biomarkers associated with POCD susceptibility. Elevated levels of circulating tau protein and neurofilament light chain serve as sensitive indicators of neuronal damage and axonal degeneration and are strongly correlated with postoperative cognitive decline (83, 84). Furthermore, inflammatory markers such as YKL-40 and IL-6 are involved in persistent neuroinflammation following surgery (84). Incorporating these biomarkers into multimodal preoperative evaluation, along with genetic profiling, could enhance risk stratification and support personalized anesthetic management.

Translating these findings into clinical practice involves addressing key challenges: (1) developing rapid, cost-effective genotyping platforms; (2) establishing evidence-based protocols for genotype-guided anesthetic selection; and (3) validating clinical utility through large-scale trials. Successful implementation will require multidisciplinary collaboration to integrate genetic profiling into routine preoperative assessment, while also addressing ethical and economic considerations.

### 4.2 Novel neuroprotective therapeutics: from bench to bedside

Current research has identified several promising pharmacological approaches targeting POCD pathogenesis. IL-1 receptor antagonists demonstrate significant anti-inflammatory effects in preclinical models, reducing neuroinflammation by 40–50% through microglial modulation (85). However, their clinical utility is constrained by limited blood–brain barrier penetration (<5% bioavailability) and potential immunosuppression risks in surgical populations (85).

Mitochondria-targeted compounds show particular therapeutic potential. MitoQ exhibits superior neuroprotection compared to conventional antioxidants, reducing hippocampal oxidative damage by 65% (*p* < 0.001) through SIRT6-mediated preservation of mitochondrial function (86). MitoQ’s unique efficacy originates from its molecular structure, in which the ubiquinone antioxidant is covalently linked to a lipophilic triphenylphosphonium cation (87). The lipophilic cation enables MitoQ’s rapid and extensive accumulation in the mitochondrial matrix, achieving concentrations several 100-fold higher than in the cytoplasm (87). This accumulation is driven by the substantial negative electrochemical potential across the inner mitochondrial membrane. Consequently, MitoQ can efficiently neutralize ROS at their primary production site. This targeted action contrasts with that of untargeted antioxidants, such as vitamins C and E, which distribute broadly throughout the cell and attain only low nanomolar concentrations within mitochondria (88). This mechanism is especially relevant for POCD given the central role of mitochondrial dysfunction in cognitive impairment.

In addition to MitoQ, several other therapeutic strategies targeting redox mechanisms are under investigation. For instance, *N*-acetylcysteine, which serves as a glutathione precursor, mitigates neuroinflammation and synaptic loss in rodent models of POCD. Its efficacy may be mediated by the suppression of the NLRP3 inflammasome (89). Similarly, the protein NUCB2/Nesfatin-1 confers neuroprotection against sevoflurane-induced cognitive deficits in preclinical models (90). Its beneficial actions involve activation of the PI3K/Akt pathway and attenuation of apoptotic cell death. Furthermore, the compound P7C3-A20 promotes hippocampal neurogenesis and preserves cognitive function following anesthesia in aged animal models (91). This neuroprotective effect is likely attributable to the direct preservation of mitochondrial membrane integrity and a concomitant reduction in apoptotic signaling.

Innovative delivery systems are addressing current limitations. Nanoparticle technologies enhance CNS drug bioavailability while minimizing systemic effects, representing a significant advancement in neurotherapeutics (92). These approaches are now being evaluated in clinical trials, which has shown promising receptor selectivity in neuropsychiatric populations (93).

Isogenic cell models, particularly those harboring mutations in genes such as APP or presenilin, serve as valuable platforms for screening compounds that mitigate anesthesia-induced amyloidogenesis and tau hyperphosphorylation (94). Furthermore, these models facilitate the dissection of specific pathogenic pathways and the validation of candidate therapeutic target engagement within a controlled genetic background (95).

Building upon these innovations, advanced delivery platforms such as exosome-based carriers offer a novel means to overcome the restrictive nature of the BBB. Exosomes exhibit intrinsic biocompatibility and have demonstrated enhanced efficiency in CNS drug delivery, enabling targeted release of neuroprotective agents with reduced systemic toxicity (96). Early-phase clinical trials in neurodegenerative diseases have shown favorable pharmacokinetics and therapeutic potential, suggesting feasibility for adaptation in POCD contexts. These findings underscore the need for translational research to evaluate exosome-mediated interventions specifically within perioperative populations.

### 4.3 Methodological considerations in POCD research

Current POCD research methodologies exhibit several critical limitations that undermine study validity. As noted by Tasbihgou et al. (97), the majority of clinical trials in this field are statistically underpowered, with sample sizes typically below 200 participants, while follow-up durations rarely extend beyond 3 months postoperatively. These constraints significantly impair the ability to detect clinically meaningful cognitive changes and establish reliable longitudinal outcomes.

The neutral findings of recent large trial, such as ENGAGES, offers important guidance for the design of future investigations (56). These results underscore that POCD and delirium are heterogeneous syndromes shaped by multiple biological and clinical pathways, rather than conditions responsive to a single, uniform intervention (56). Consequently, future trials should move beyond isolated intraoperative variables and instead evaluate multicomponent perioperative intervention bundles that span the full surgical continuum. Such bundles may include prehabilitation programs that enhance cognitive, physical, and nutritional resilience; multimodal intraoperative monitoring, such as depth of anesthesia assessment, cerebral oximetry, and goal-directed hemodynamic therapy; and standardized postoperative care protocols designed to reduce delirium triggers through sleep promotion, early mobilization, and optimized analgesia.

Future POCD trials should address disease heterogeneity by replacing universal treatment strategies with precision enrichment approaches. Targeting high-risk patients—identified through genetic markers (e.g., ApoE ε4), biomarkers (e.g., neurofilament light chain), or clinical risk scores—enhances statistical power and improves detection of meaningful treatment effects in defined cohorts (79, 83, 84). We contend that demonstrating efficacy in unselected populations is inherently problematic, as interventions may only benefit specific pathophysiological subtypes. This precision framework aligns with the “two-hit” model of POCD and provides the methodological rigor necessary for evaluating neuroprotective therapies. Ultimately, advancing clinical practice requires shifting from universal solutions toward personalized perioperative neuroprotection bundles.

Standardized assessment protocols are essential for advancing POCD research. Wang et al. emphasize the importance of comprehensive neuropsychological batteries administered at multiple time points, coupled with advanced neuroimaging techniques (2). Such multimodal approaches provide both sensitive cognitive measures and objective biomarkers of neural dysfunction, addressing the historical reliance on inconsistent assessment methodologies.

Recent developments highlight promising solutions to these methodological challenges. Zhao et al. document the benefits of international research consortia in establishing standardized protocols and facilitating large-scale studies (98). These collaborative efforts, combined with emerging digital assessment technologies, offer new opportunities to overcome previous limitations in sample size, follow-up duration, and data consistency while maintaining rigorous scientific standards.

To address challenges related to small sample sizes and inconsistent methodologies, future POCD research should implement standardized multicenter clinical trial frameworks. Collaborative networks among academic centers and regional hospitals can enable standardized recruitment using uniform eligibility criteria and validated cognitive assessment tools (99). Consistent follow-up intervals (e.g., baseline, postoperative days 7 and 90, and 12 months) would enhance comparability of longitudinal data. Centralized data platforms and federated analytical frameworks would support robust statistical analysis while maintaining data privacy and site autonomy.

These mechanistic disturbances collectively underlie the diverse clinical manifestations of POCD. Microglia-driven neuroinflammation primarily underlies acute attention and executive dysfunction; mitochondrial metabolic failure contributes to slowed processing speed and impaired learning; and impaired synaptic remodeling and neurogenesis underlie prolonged memory deficits, particularly in vulnerable older adults. This integrated framework bridges mechanistic insights with clinical phenotypes and accounts for the limited efficacy of interventions targeting isolated intraoperative factors. Consequently, future research should prioritize stratifying patients by their predominant mechanisms for aligning therapeutic strategies with specific cognitive phenotypes.

## 5 Summary

In summary, this perspective analyses anesthesia’s role in POCD pathogenesis through the unifying “two-hit” model, wherein perioperative insults amplify pre-existing neural vulnerability. We have integrated evidence highlighting three interconnected mechanistic pillars: neuroinflammatory activation, mitochondrial dysfunction with oxidative injury, and impaired synaptic plasticity. Central to our analysis is that the synergistic interaction among these pathways—not their isolated effects—drives the cognitive impairment characteristic of POCD. This synthesis further demonstrates that anesthesia contributes to POCD through three established pathways—microglia-mediated neuroinflammatory activation, disturbances in cerebral hemodynamics and metabolic homeostasis, and direct impairment of synaptic plasticity and neurogenesis. Together, these mechanisms produce a neurobiological milieu that promotes cognitive impairment extending beyond the immediate postoperative period.

Evidence-based preventive strategies include propofol-based total intravenous anesthesia when appropriate, BIS-guided depth-of-anesthesia monitoring, and regional anesthetic techniques, all of which have been associated with a 25–40% reduction in POCD incidence. These approaches limit unnecessary anesthetic exposure while maintaining adequate surgical conditions, providing particular benefit to older adults and patients at elevated risk.

Three priority directions emerge: development of validated biomarkers for preoperative risk stratification, incorporation of pharmacogenomic tools to individualize anesthetic selection, and optimization of neuroprotective agents with improved blood–brain barrier penetration. Successful translation will require multicenter studies employing standardized cognitive assessment batteries and extended follow-up intervals. Integrating current evidence into clinical practice and pursuing focused investigation of these priority areas will substantially advance POCD prevention and management. This dual approach addresses immediate clinical needs while simultaneously targeting key knowledge gaps, offering significant potential to reduce the individual and societal burden of perioperative cognitive disorders.

## Data Availability

The original contributions presented in the study are included in the article/Supplementary material, further inquiries can be directed to the corresponding author/s.
