# Supplementary material for: Anesthesia-related factors in the pathogenesis of postoperative cognitive dysfunction: a mechanistic perspective
Source: Front Neurol. 2026 Jan 5;16:1700911. doi: 10.3389/fneur.2025.1700911 (PMC12812579; doi:10.3389/fneur.2025.1700911)
Supplement: Supplementary file 1 [file Table_1.docx]

**Supplementary Table S1. Comparison of anesthetic approaches, mechanistic effects, and postoperative cognitive outcomes**

| Anesthetic approach | Mechanistic effects (neuroprotective or neurotoxic) | Clinical cognitive outcomes | Supporting evidence |
| --- | --- | --- | --- |
| Volatile anesthetics (sevoflurane, isoflurane) | ↑ Microglial activation; ↑ IL-6, TNF-α; ↑ β-amyloid aggregation; ↓ neurogenesis | 15–25% higher POCD incidence in elderly; worsened early cognitive scores | Pang et al., 2021; preclinical neuroinflammation studies |
| Propofol-based TIVA | ↓ Neuroinflammation; preserved synaptic plasticity; high doses impair mitochondria | Lower POCD incidence vs volatile (day 7); stable long-term recovery | Radtke et al., 2013; meta-analyses |
| Benzodiazepine-adjunct GA | Excessive GABAergic inhibition; network desynchronization; mild anticholinergic effects | ↑ Risk of delirium; unclear direct POCD linkage | AGS guidelines; delirium-focused trials |
| Dexmedetomidine-adjunct GA | ↓ Neuroinflammation; α2-mediated microglial suppression; preserved cerebral blood flow | 30–40% reduction in early POCD; improved postoperative trajectories | Deiner et al., 2017; meta-analyses |
| RA | ↓ Systemic inflammatory response; ↓ opioid load; minimal direct CNS effect | No major difference vs GA in large RCTs (RAGA) | RAGA trial; systematic reviews |
| Multimodal anesthesia/analgesia | ↓ Opioid exposure; ↓ cytokine surge; ↓ BBB disruption; improved sleep architecture | 20–30% improvement in attention and memory at 3 months | Multimodal analgesia trials; NSAID models |
| Cerebral-protective monitoring (BIS, NIRS) | Avoids burst suppression; maintains cerebral perfusion; ↓ hypoxia-induced injury | ↓ Delirium and POCD when preventing deep anesthesia or desaturation | ENGAGES EEG studies; NIRS trials |

**Notes**: POCD, postoperative cognitive dysfunction； IL-6, interleukin-6； TNF-α, tumor necrosis factor-alpha； TIVA, total intravenous anesthesia； GA, general anesthesia； RA, regional anesthesia； CNS, central nervous system； BBB, blood-brain barrier； NSAID, non-steroidal anti-inflammatory drug； BIS, bispectral index； NIRS, near-infrared spectroscopy； RCT, randomized controlled trial.
